# The Role of Clinical Researchers During COVID-19: Balancing Individual, Scientific, and Social Benefits of Research

**DOI:** 10.3389/fpubh.2021.638964

**Published:** 2021-04-07

**Authors:** Diana C. Oviedo, Ambar R. Perez-Lao, Alcibiades E. Villarreal, Maria B. Carreira, Gabrielle B. Britton

**Affiliations:** ^1^Escuela de Psicología, Facultad de Ciencias Sociales, Universidad Católica Santa María La Antigua (USMA), Panama City, Panama; ^2^Centro de Neurociencias y Unidad de Investigación Clínica, Instituto de Investigaciones Científicas y Servicios de Alta Tecnología (INDICASAT AIP), Panama City, Panama

**Keywords:** clinical research, COVID-19, bioethics, psychological distress, death and mourning

## Introduction

Clinical and research psychologists around the world are experiencing various challenges due to the COVID-19 pandemic. Quarantine, mobility restrictions and health risks associated with the new SARS-CoV-2 virus have disrupted studies, which has impacted data collection, project coordination and monitoring efforts. Researchers have had to shift and adapt their fields of research. Consequently, various studies regarding COVID-19 have emerged. In Panama, a multidisciplinary research group, the Panama Aging Research Initiative (PARI), has been studying the characteristics associated with aging among the Panamanian population for the last 10 years. Due to the COVID-19 pandemic, enrollment and assessment of elderly participants came to a halt being as they are most vulnerable to COVID-19. As the team became involved in pandemic-related studies, it faced an unfamiliar challenge: to collect data from hospitalized patients who had tested positive to SARS-CoV-2. This opinion article aims to present our experience with COVID-19 patients and critically explore the role of clinical researchers in emergency situations as they balance between the individual, scientific and social benefits of research.

### Ethical Issues During Public Health Emergency Situations

Conducting research during health emergency situations is an ethical responsibility for researchers, institutions and countries (1). From vaccine and pharmaceutical clinical trials to psychology and social research, obtaining scientific data is critical to create guidelines, adequately clarify or identify risk factors and clinical symptoms, evaluate tests and generate appropriate interventions (2). Nevertheless, collecting clinical data in emergencies requires adaptation to extreme settings, flexibility, and agility (3, 4). Health emergencies imply special ethical circumstances above and beyond normal.

Initially, protocols must undergo evaluation and approval from a bioethics committee. Nevertheless, in some countries ethic reviews can take months; therefore, in emergencies it is crucial that institutions accelerate review processes of research protocols while maintaining quality (1, 5). Also, in health crises, the perception of risks and benefits must be taken into account, as these can change over time. Moreover, accountability and transparency must be carefully monitored (5). Informed consents as well as other ethics considerations such as ensuring ethical treatment of vulnerable groups, guaranteeing scientific validity and social value, benefit vs. risks assessments, are fundamental in researching during critical conditions (6).

### Sociocultural and Socioeconomic Considerations for Research in Low- and Middle-Income Countries

Conducting research in health emergencies poses many challenges, particularly for low- and middle-income countries. These obstacles can include the unpredictable nature of the crisis itself, limited healthcare systems and infrastructures, ruptured communication between the scientific and political systems, limited funding, and inadequate policies in response to epidemics (1). In Asian and African countries, previous pandemics such as Ebola, SARS-CoV1 and MERS-CoV gave researchers an idea of what to expect during COVID-19 pandemic (7, 8). Formerly, in the Latin American and Caribbean (LAC) region, a Zika outbreak forced researchers to generate research networks rapidly in order to be able to respond to the different needs as the outbreak unraveled (9). Nevertheless, the LAC region was unprepared for the current pandemic's magnitude and has become one of the most affected regions (10). In the case of Panama, as in most countries, COVID-19 revealed many social, health, economic and educational inequalities and has mainly affected the most disadvantaged individuals (10). Data collection in the hospital research context revealed insufficient medical personnel and infrastructure. Also, researchers encountered complex challenges in enrollment, such as difficulties reaching patients eligible for the study, as well as participants' fears and psychological distress.

## Psychological and Social Impact of COVID-19

In Panama, in April 2020, the PARI group began a seroprevalence antibody study in three different groups, namely healthcare workers, healthy controls recruited from a blood donor clinic and SARS-CoV-2 positive hospitalized patients from public hospitals (11). The research instruments included an initial informed consent, an interview to obtain sociodemographic information, previous diseases and COVID-19 related symptoms and the collection of a blood sample. Data collection in the current health emergency, specifically from positive hospitalized patients posed a series of adversities. As it was the beginning of the pandemic, different situations hindered research conditions; knowledge on the virus was scarce, there was a high mortality rate and there were no approved treatments. Additionally, as in many countries, we faced situations such as limited personal protection equipment and poor conditions in hospital facilities.

In this high uncertainty, high risk context, we also faced limitations regarding participant selection. Patients' level of illness varied. Some patients were delirious, cognitively impaired or experiencing psychological distress affecting their ability to talk. This required a careful evaluation of which participants were eligible to be offered participation in the study. We had to seek the balance between being just and offering the study to everyone, but also recognizing whether or not some people in a situation of vulnerability can be contemplated as research participants.

Second, as we addressed the COVID-19 patients, we were faced with realities that included educational, cultural and language barriers. Such challenges are common to many studies in Panama. One of the main difficulties was a low literacy level. Even though literacy rates in the last 12 years have increased in 50% and currently adult literacy rates are ~95.4%, the mean number of years of schooling is 10 (12) and education quality has been reported to be deficient (13). Moreover, some of the patients belonged to indigenous groups, therefore we had to take into consideration culturally appropriate materials.

Third, some of the other patients who understood the study and signed the informed consent viewed their participation as an opportunity to talk about the deficiencies they experienced at the hospital, such as the conditions of the rooms, bathrooms, food and the understaffed hospital wards. Although these situations are not research limitations *per se*, they can contribute to the psychological burden patients sustain.

Fourth, one of the biggest challenges we confronted conducting our study was the impact of COVID-19 on mental health. As we collected data, we had to consider that a large portion of SARS-CoV-2 positive participants were under extreme stress and fear. Topics such as loneliness, uncertainty, confusion, anger, sadness, anxiety, and stress were often discussed among patients. Literature has shown that symptoms of psychological distress, are associated to hospitalizations (14, 15). Isolation and quarantine where patients are not allowed to receive visitors often augment these psychological and psychiatric symptoms (14, 16). In addition to this, healthcare professionals have to deal with an overload of patients and work, often limiting the time they can spend with each patient aggravating the loneliness and despair patients experience.

Lastly, a recurring fear manifested by most patients, independent of their disease severity, was that of their own death. Moreover, others had witnessed other patients in their rooms dying; and a patient even had to intervene in a suicide attempt. Lastly some of the interviewed patients had been admitted to the hospital with a family member, and while hospitalized, their loved one passed away. Psychological distress regarding death in hospitalization situations and associated to pandemics and epidemics has been previously studied (14–17). From mourning to fear of dying, death is an extremely relevant topic that must be taken into account when approaching hospitalized patients. Studies have shown that in patients who recover from life threatening diseases, the experience of being hospitalized is associated with post-traumatic stress disorder and can be highly intensified by grief (15, 18). Having all this in mind, we had to rapidly assess if answering questions that were related to participant's health contemplated in our study, would emotionally and psychologically harm them.

As patients discussed the anguish they had experienced after contracting the virus, some of them evidenced the coping mechanisms they had developed through their convalescence. Some mentioned they had turned to faith and were constantly praying and thanking God for being alive and this helped them maintain optimistic. Others, turned to their roommates looking for comfort in their new friendship. Evidence suggests that, as witnessed, often patients look for external mechanisms such as spirituality and religion, gratitude, and social support to help them cope with burdensome situations (16, 19).

## Conclusions

Conducting research during public health emergencies demands an adequate balance of social, scientific and individual benefits. Researcher's roles in clinical settings during COVID-19 require a comprehensive understanding of ethical principles and an empathic engagement with participants (6). Ethical considerations are fundamental from the conception and planification of the study, to the actual field work of data collection, publishing and sharing of results (2). As we conducted our study, we constantly asked ourselves, how do we draw the line between benefits for science, participants and knowledge?

At an individual level, we had a duty to always seek benefits for participants. A critical analysis had to be made regarding possible psychological or social harms of the study, as well as acknowledging and empathizing with patients' vulnerable states. Additionally, we needed to make sure they understood the study and made a voluntary decision to participate.

At a scientific level, due to the complexity, novelty and unexpectedness of COVID-19, we as other researchers around the world, have urgently responded by rapidly generating data while maintaining scientific validity and replicability. Researchers and work groups have had to generate multiple therapeutic strategies, prevention mechanisms and diagnostic tests to tackle this new disease. Moreover, the COVID-19 pandemic has exposed the importance of research's social benefits. Knowledge cannot be limited to a laboratory or to a publication. It is mandatory that research in this health emergency has practical applications that rapidly reaches all countries affected by the virus. In the case of the PARI COVID-19 study, over the last months there has been an important increase in the use of the antibody test.

As a multidisciplinary group we have engaged in multiple areas of science. Our previous experience in research with elderly population, aided us in conducting the COVID-19 project as it gave us tools to work and assess vulnerable groups. In our aging study, some participants have physical and cognitive impairments, frailty, a limited functional status as well as low literacy levels and/or economic limitations. Therefore, as researchers we are obliged to acknowledge their vulnerability and carefully ensure all ethical processes are taken into account.

The current study has shown us the immense need to address the long term psychological and social effects of the COVID-19 pandemic. Even though more than 80% of patients will recover from the virus, the pandemic will continue to have a detrimental mental health burden on various population groups (20). Therefore, as clinical and psychology researchers, we recommend the following:

1. Creating research platforms dedicated to generating knowledge, using open data and aiding in the management of mental health issues. To ensure the creation of such platforms, investment in research must be a priority.
2. As scientific data is generated, it is relevant to divulge scientific findings in a simple way. Science illiteracy even among educated population poses a challenge, especially in the context of widespread conspiracy theories and fake news.
3. It is imperative to include mental health as part of countries' response plans, which includes an increase in funding and the promotion of policies that support efficient mental health services.
4. Finally, in hospital contexts, we recommend the generation and use of liaison psychiatry, allowing more mental health professionals to attend COVID-19 patients while being hospitalized. Currently, psychologists and psychiatrists become involved when there is a crisis situation with a particular patient, nevertheless, continuous mental health assistance is greatly required.

It's time to move beyond the initial critical sanitary response to a sustainable global effort toward resilience.

## Author Contributions

DO conceived and wrote the manuscript. GB, AV, AP-L, and MC read, reviewed and equally contributed to the intellectual content, and format of the manuscript. All authors approved the submitted version.

## Conflict of Interest

The authors declare that the research was conducted in the absence of any commercial or financial relationships that could be construed as a potential conflict of interest.
